# IOTA-Based Distributed Ledger in the Mining Industry: Efficiency, Sustainability and Transparency

**DOI:** 10.3390/s24030923

**Published:** 2024-01-31

**Authors:** Nenad Gligoric, David Escuín, Lorena Polo, Angelos Amditis, Tasos Georgakopoulos, Alberto Fraile

**Affiliations:** 1 Zentrix Lab, Blockchain Development Department, Milosa Trebinjca 10, 26000 Pancevo, Serbia; 2 ITAINNOVA—Instituto Tecnológico de Aragón, C. María de Luna, 7, 50018 Zaragoza, Spain; descuin@itainnova.es (D.E.); lpolo@itainnova.es (L.P.); 3 Institute of Communications and Computer Systems: ICCS, 28is Oktovriou 42, 106 82 Athina, Greece; a.amditis@iccs.gr (A.A.); a.georgakopoulos@iccs.gr (T.G.); 4 Escuela Superior de Ingeniería y Tecnología (ESIT), Universidad Internacional de La Rioja (UNIR), 26006 Logroño, Spain; alberto.fraile@unir.net

**Keywords:** blockchain, IOTA, industry 4.0, mining, Internet of Things, trust

## Abstract

The paper presents a traceability framework founded upon a methodological approach specifically designed for the integration of the IOTA-based distributed ledger within the mining industry. This framework constitutes an initial stride towards the certification and labelling of sustainable material production. The efficacy of this methodology is subject to real-world evaluation within the framework of the European Commission funded project DIG_IT. Within the architectural framework, the integration of decentralized identifiers (DIDs) and the IOTA network are instrumental in effecting the encryption of data records, with associated hashes securely anchored on the explorer. Recorded environmental parameters, encompassing metrics such as pH level, turbidity, electrical conductivity, and emissions, serve as tangible evidence affirming their adherence to prevailing regulatory standards. The overarching system architecture encompasses a sophisticated Industrial Internet of Things platform (IIoTp), facilitating the seamless connection of data from a diverse array of sensors. End users, including governmental entities, mining managers, and the general public, stand to derive substantial benefits from tailored dashboards designed to facilitate the validation of data for emission compliance.

## 1. Introduction

The transparency of the processes and innovation culture in the mining industry is a challenge. Even when the data about certain operations and environmental pollution are presented, proving to the authorities and local community that the data recorded are true and have not been changed is difficult. The listing of a company on an international stock exchange does not ensure a high level of environmental transparency, as asserted in [1]. Additionally, Ref. [2] identifies a significant deficiency in terms of entities’ adherence to the legal framework, wherein web pages seem more tailored for shareholders and investors than for the affected communities.

The main contribution of this paper is to present a novel approach for ensuring data integrity and public auditability in sensors and detectors in the mining industry. The provenance of data recorded through such a system will ensure that the data are immutable and traceable. To the best of current knowledge, this is the first attempt to define the methodology and to evaluate the approach in a live environment. The utilization of the IOTA Tangle (a distributed ledger technology, DLT) as a verifiable data registry has proven to be effective for security control purposes.

This paper describes the innovation challenge conducted to enhance traceability and accountability within the mining industry through blockchain technology. Our primary objective does not encompass the active pursuit of new blockchain consensus algorithms. Instead, our focus lies in aiding the mining industry to attain heightened levels of transparency and efficiency in line with their organizational objectives. Even though Industry 4.0 sensors offer a wide spectrum of connectivity solutions, data acquisition, and intelligent technologies to address some challenges, the mining industry is hesitantly moving towards adopting innovative approaches when compared to downstream industries. The approach has been validated within the DIG_IT [3] project funded by the European Commission that aims to address the sustainable use of resources by developing technology for the monitoring of mining operations as a first step of certification for labelling sustainable material extraction. The mines included in the project cover a range of operations, encompassing underground quarrying in Italy, as well as open-pit mining in Spain, Norway, and Finland.

However, it is not only transparency and sustainability that are crucial. Compliance with the General Data Protection Regulation (GDPR), along with technically feasible autonomous monitoring, are essential for ensuring the sustainable extraction of raw materials in the mining industry. More specifically, the methodology proposed in this manuscript for traceability in mining industry contributes to the global-scale digitalization of mining operations that it is estimated to generate an annual reduction of 61 million tonnes of CO_2_ emissions according to the World Economic Forum.

The structure of this paper is organized as follows: Section 2 provides an examination of related work, offering an exploration of ongoing research within the existing literature. The definition of labelling sustainable material certification is revealed in Section 3, while Section 4 describes the DLT-based system architecture, elucidating issues pertaining to data collection and encryption mechanisms designed to ensure data integrity and confidentiality. The explication of the platform architecture is also encompassed within this section. Section 5 describes the system dashboards, providing a detailed account of their composition and functionalities. Subsequently, Section 6 engages in a comprehensive discussion of the broader implications of the innovation and outlines potential avenues for future research. Concluding remarks are encapsulated in Section 7.

## 2. Related Work

Raw material extraction is essential to meeting the growing demands of industrialisation and technological advancement. These raw materials, such as metals, minerals, and fossil fuels, form the backbone of modern economies, powering various sectors like construction, manufacturing, energy production, and transportation. They are crucial for the production of goods and infrastructure, making them indispensable for economic development and societal progress. However, sustainable and responsible extraction practices are increasingly important to balance resource availability with environmental conservation and social considerations.

In addition, the escalating need for a wide range of raw materials, including lithium, cobalt, nickel, rare earth elements, and copper, has been catalysed by the global pursuit of carbon neutrality [4]. The extraction of these raw materials plays a pivotal role in enabling the transition to low-carbon technologies. For instance, lithium-ion batteries, which heavily rely on lithium, cobalt, and nickel, power electric vehicles and store renewable energy, ensuring grid stability and sustainability [5]. The heightened demand for these critical minerals has led to a significant upswing in mining activities [6,7], and now, the sustainable sourcing and responsible extraction of these raw materials has become imperative [8]. Transparent disclosure of mining practices, environmental impact assessments, and social responsibility initiatives will hold mining companies accountable for their actions [9]. By embracing process transparency, the mining industry can mitigate the negative impacts of raw material extraction, foster responsible mining practices, and contribute to global carbon neutrality goals [10,11]. This requires new methods and approaches that enable efficient monitoring of the mining industry, ensuring process transparency to address the challenges effectively.

This section reviews the domain of effective monitoring of environmental sustainability in the mining industry by using DLT or blockchain. While most of the blockchain papers strive to move to more energy and price efficient algorithms, the application of DLTs in real-world use cases for the sustainable mining industry is very scarce. The scalability and energy consumption are among the foremost concerns, as blockchain networks, particularly proof-of-work-based systems, can be resource-intensive [12].

In [13], the authors suggest using blockchain to impose the sustainable management of natural resources in two use cases: deforestation and groundwater management. The paper proposes a tokenized approach with incentives provided to land managers for changing land cover to forest, or for maintaining forest. This study describes the blockchain infrastructure without going into the technical details, deployment issues, and security implications, which could be a main point of failure in data traceability and integrity. Similar tokenisation-incentivised models are examined in [14].

In [15], a system architecture for mining machine inspections using off-the-shelf mobile devices and integrating IoT and blockchain technologies is proposed. This study is very relevant to this research, but the proposed approach is focused on the collection of data from the inspector carrying the device, considered to be a trusted source of data. This means that real-time monitoring is not possible, except in cases when the inspector is performing in field measurements.

Blockchain research and application in raw material extraction for mining are underdeveloped in comparison to other supply chain traceability technologies, such as textile [16], food [17], pharma [18]. Currently, most of the research and policy work is focused on the analysis of how DLTs can counter specific supply chain and operations management challenges [19,20]. The Organisation for Economic Co-operation and Development (OECD) reports provide recommendations and due diligence guidance for a responsible supply chain of minerals [21].

With regard to blockchain-based traceability systems, many papers can be found in the literature. The paper presented in [22] describes a traceability system for the storage and query of product information in a supply chain of agricultural products. The authors provide performance analysis and practical application. The results show that the system improves the query efficiency and security of private information, guarantees the authenticity and reliability of data in supply chain management, and meets actual application requirements. In [23], authors present an efficient traceability system for managing products in the fishery supply chain. Negligence in products’ traceability can result in food fraud that may adversely affect a consumer’s health.

In subsequent sections of the paper, an exhaustive account of the rationale behind the selection of DLT will be meticulously detailed, specifically elucidating the considerations that propelled the adoption of the IOTA network. Benefits of the usage of IOTA ledger can be found in [24]. Here, authors highlight the main features of the IOTA 2.0. the initial version of IOTA Tangle faced issues with centralization and scalability. IOTA 2.0 addresses these by removing the centralized coordinator and introducing improvements to enhance decentralization and scalability, along with providing a technical overview and future research directions for IoT applications. The work presented in [25] leverages IOTA Tangle as part of microgrid transactive energy systems, demonstrating that this technology can be cost-effective for any domain.

In conclusion, the utilization of blockchain-based systems permits the meticulous recording and tracking of each stage in the mineral production chain, thereby guaranteeing the ethical sourcing of minerals and their freedom from association with conflict zones. However, the practical implementation of such approaches remains deficient, with an insufficient number of deployments to warrant consideration as an established practice. The system posited in this paper not only furnishes valuable services for end users but also holds the potential for extension to other applications within the mining domain. 

## 3. Labelling Sustainable Material Certification

In the mining industry, ensuring the integrity and authenticity of reported data, particularly pertaining to emissions and environmental parameters, is of utmost importance for regulatory compliance and transparency. Regulatory bodies are verifiers requiring mining companies, the provers, to maintain an immutable record of their daily emissions, suspended metals, water levels, pH, and related factors. This record must be securely stored within the company’s infrastructure while also being readily accessible for government scrutiny as well as to local communities over the public (permissionless) DLT. A key requirement is to demonstrate that the data have not been tampered with since their initial reporting. Having said this, the use case in the mining industry that this paper addresses is related to labelling sustainable material certification.

This use case covers a diverse array of mines, each with its unique set of goals and challenges. From underground quarries to expansive open-pit mines, these case studies represent excellent field study for performance evaluation of the proposed approach. The mines included are Marini Marmi in Italy (underground quarry), La Parrilla mine in Spain (open pit), Titania in Norway (open pit), Hannukainen mine in Finland (open pit), and Sotkamo Silver mine in Finland.

The transparency and public accountability of the mining industry can be further enhanced by making the data (represented by a unique message ID) accessible through public dashboards. This ensures that stakeholders, including the general public, can monitor the environmental impact of mining operations. Additionally, verifiers, representing the government agency or citizens, can seamlessly access the message ID provided by provers. This message ID should serve as a direct link to the DLT explorer, a tool that facilitates the verification process by offering an immutable and transparent trail of data provenance.

**Figure 1:**
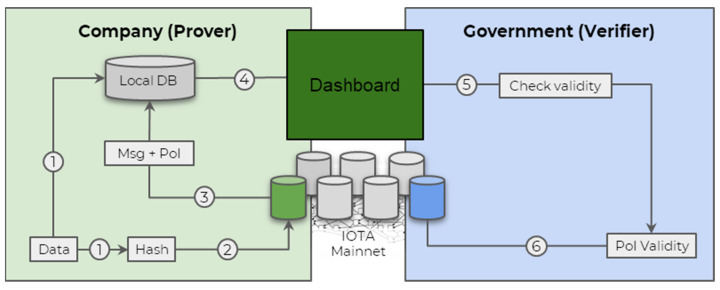
Diagram of labelling certification for sustainable mining. In the figure, IOTA Mainnet represents the DLT.

To better illustrate this concept, let us consider the following scenario (Figure 1). A government (verifier) demands mining companies (prover) to log their daily emissions (suspended metals, metals, water level, pH, etc.) in an immutable way. The data can remain with the company, but they need to be able to show it to the government at any point in time and prove it has not been tampered with since its reporting day. The data are displayed or accessible using a user-friendly way such as a dashboard, and the verifier should be able to access the message ID from the prover that leads straight to the DLT explorer. Otherwise, the prover could attach multiple results in the same day and pick the “best fitting” one when being audited.

The quality and performance data of the enterprises should be considered private and will only be available after granting permission. This being said, where possible and where there are no particular ethical concerns (biometric and personal data from workers, for instance), monitoring data generated and collected during the auditing should be freely distributed.

## 4. DLT-Based System Architecture

In this section, the high-level system architecture to address the challenge of sustainable monitoring is elucidated. Here, technical and non-technical components are presented together with a methodological approach of data collection and preservation in the DLT network.

In the modern landscape of mining operations, data collection plays a pivotal role in enhancing efficiency, safety, and overall productivity. Mines, whether surface or underground, are complex ecosystems where a multitude of variables interact dynamically. The extraction of valuable resources, the monitoring of equipment health, the assessment of environmental impacts, and the safeguarding of personnel all demand a comprehensive understanding of real-time conditions. The proposed architecture gathers all those features and has already transitioned to the production stage.

### 4.1. Data Collection

**Figure 2:**
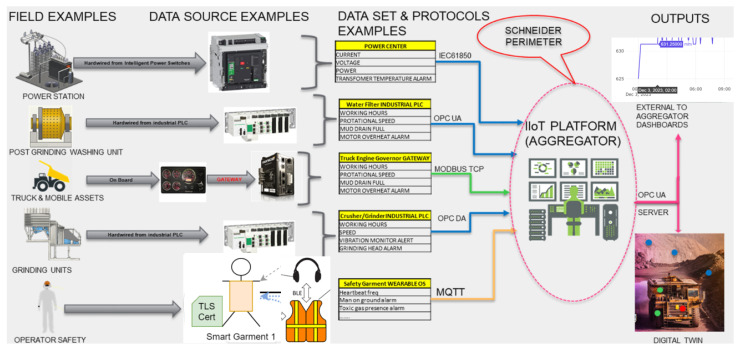
DIG_IT platform data collection from environment, humans and assets.

The data collection within mining operations uses diverse methodologies and technologies employed to capture, transmit, and analyse crucial information. The IIoTp is designed to improve the efficiency and sustainability of mining operations by connecting cyber and physical systems. The platform collects data from sensors at 3 levels: human, assets, environment, and will also incorporate both market real-time and historical data (Figure 2).

- Field Assets: These are physical assets or locations where operations are conducted or monitored, like a power station, post grinding washing unit, truck and mobile assets, grinding units, and considerations for operator safety.
- Data Source Controllers: These represent hardware devices that gather data from the field assets. They include intelligent power switches, industrial programmable logic controllers (PLCs), gateways for mobile assets, and wearable devices for safety monitoring.
- Dataset and Protocols Examples: The middle section details the kind of data collected (like current, voltage, power, temperature alarms, etc.) and the standard communication protocols used to transmit these data to the IIoT platform.
- Schneider Perimeter: The outlined IIoT ecosystem is within the scope of Schneider Electric’s solutions, products, or services. This is one of the project partners and provides electronic devices and hardware.
- IIoT Platform (Aggregator): Software solution that aggregates the data from various sources, processes it, and may also allow for control commands to be sent back to the field assets. It is represented as the central system where all data converges.
- Outputs: On the right, the outputs of the IIoT platform are shown. This includes dashboards for data visualization (see Section 5), servers for data processing and storage, and a digital twin, which is a virtual representation of the physical assets, allowing for simulation, analysis, and control.

### 4.2. Security and Encryption

The blockchain traceability is achieved by hashing a specific sensor dataset. Hashing is a cryptographic one-way function that creates a digital fingerprint of defined length from an arbitrary dataset. The same hash algorithm will always lead to the same hash output for the same input data. A data hash stored on a blockchain provides just as many guarantees in terms of data immutability as if the actual dataset itself is stored. The hash does not, however, give away any information since it cannot be inverted to reproduce its original data. The result of the hashing operation at any given time could be then compared with the retroactive hash saved during the creation of the dataset. Thus, it is of the highest importance that the source is trusted and that, in the case of the industry mining use case, the security between the data source or the data provider and the DLT is encrypted, robust, and can be trusted. In order to comply privacy regulation to the GDPR, any data that ends up in the blockchain must be anonymised before the hash of the data is created and recorded on DLT.

Based on the actual use case requirements and if the sensor data are sensitive or not, encryption is also used to encrypt datasets. By applying advanced encryption techniques, raw data collected from mining operations can be transformed into encrypted formats before being stored on the DLT. This ensures that even if unauthorised parties gain access to the data, they are unable to decipher its contents without the corresponding decryption keys.

The following section will discuss the selection of the DLT and its associated security aspects, particularly focusing on susceptibility to various attacks. This analysis aims to provide an understanding of the vulnerabilities inherent in DLT systems and the measures taken to mitigate such risks.

### 4.3. Choice of DLT

There are a number of different DLT networks that can be used to achieve traceability of the data. Permissioned DLTs (such as Hyperledger Fabric) were not considered simply because they are in consortium and the trust necessary to be established in the mining industry explicitly requires public audit.

The choice of DLT depends on the desired level of decentralisation, scalability, security, and energy efficiency. These factors are mainly influenced by the consensus mechanism that the specific DLT is using. For instance, the proof-of-work (PoW) has high security due to the computational power required to mine blocks, but it consumes a significant amount of energy and has slower transactions, and thus there are processing and scalability concerns that can affect real-time traceability. The main protocols based on PoW are Bitcoin, Litecoin, Ethereum 1.0, etc. Currently, Ethereum has shifted to PoS in its latest version.

The proof-of-stake (PoS) consensus mechanism consumes less energy, making it more sustainable, and it has faster transactions that can improve traceability and data handling speed in comparison to PoW. There are centralisation risks due to the existence of actors in the network with more influence, and it is less secure. There are still gas fees for writing the data but they are less expensive when used to store the large amounts of data in comparison to PoW-based blockchain. The main PoS blockchains are Ethereum 2.0 (Polygon as layer two of Ethereum), Polkadot, Avalanche, Cardano, Solana, etc. Of course, there are more ledgers, but these are the most popular ones with most stable user and transaction base.

Delegated proof-of-stake (DpoS) is a consensus mechanism commonly used in blockchain networks where token holders vote to elect a limited number of delegates or validators who are responsible for validating transactions and producing blocks. DpoS aims to improve scalability and energy efficiency compared to traditional PoW networks. There are also a number of interesting DpoS-based DLTs such as 0Bsnetwork and EOSIO.

Blockchains based on the proof-of-authority (PoA) consensus mechanism offer high transaction throughput for efficient traceability, but the network relies on a limited number of approved validators, which means they are not fully decentralised. Another interesting consensus algorithm is presented in [26]. Here, the authors describe a blockchain consensus mechanism tailored to support mathematical optimization problems, called proof-of-solution (PoSo). PoSo differs from PoW in that it replaces the meaningless mathematical puzzle with a meaningful optimization problem

In the DLT ecosystem, there are also other algorithms such as directed acyclic graph (DAG), offering high throughput for traceability applications and lower fees. Implementing and understanding the DAG can be complex, and the DAG-based DLT can face different security challenges compared to traditional blockchains. Furthermore, DAG has a very-high energy efficiency, and the current IOTA protocol has simplified the integration complexity by providing L2 (layer two) frameworks that help implement different services on top of L1 (layer one, which is a core protocol).

In Table 1, a comparison of features is given for different DLTs taking into account supported Transactions Per Second (TPS), price, support for DID (explained below) on L1 or L2, as well as the hardware library support for devices such as the main metrics. The values are taken from relevant research papers and relevant online sources. The below metric cannot be used as comparative performance evaluation, as it would need to have the same data written in different networks and as gas price is continually changing—repeating the same transaction may result in different transaction fees, which why this analysis was conducted to justify the selection of a DLT used to implement the proposed approach. The transaction fee was calculated by multiplying the sum of the gas price and the current value of the token in dollar/ euro.

The reference number of minimum 1000 TPS was taken using the message count from the DIG_IT project. This number can be significantly higher depending on the number of assets, sensors, and processes monitored. At its best, the DLT should support as many TPS as possible with minimal fees, it should have an integrated DID framework, and it should have support for the embedded devices. From Table 1, IOTA and EOSIO are the best candidates when taking into account the set criteria. Specifically, the IOTA protocol encompasses an (L2) framework, facilitating the seamless integration of DIDs. It distinguishes itself by refraining from imposing fees for data inscription in the DLT, incorporates encryption schemes such as the Streams protocol for data anchored on the IOTA network, and exhibits notable energy efficiency. This comprehensive suite of features positions the IOTA protocol as a judicious choice in meeting the multifaceted demands of the envisioned methodology.

As highlighted in [27], the choice of DLT necessitates the exploration and clarification of potential security risks. The IOTA Tangle was also chosen for its robust defence against security threats such as double-spending and Sybil attacks.

- Double-spending in IOTA: Double-spending is a risk in digital currencies where the same digital token can be spent more than once. This is a significant concern in blockchain technologies. In the IOTA Tangle, this risk is mitigated because as a transaction receives more confirmations, it becomes increasingly trusted. For the specific use case of labelling sustainable material certification, double-spending is not relevant as it does not involve currency transactions. This indicates that the IOTA’s approach is well-suited for non-monetary applications where the integrity of data, rather than currency, is paramount.
- Sybil attack and IOTA’s defence: A Sybil attack, where an attacker creates multiple fake identities to influence a network, is addressed by the IOTA’s unique system. In the IOTA, nodes maintain a list of trusted entities, which adds a layer of security against such attacks. For an attacker to compromise the system, they would need to corrupt a majority of these trusted entities, a feat that becomes increasingly difficult as more users join and decentralize the network. Furthermore, the flexibility of the IOTA’s system to edit the list of trusted entities and the ability to revert individual transactions under consensus adds to its resilience.

For the application of mining traceability, the IOTA’s features are particularly advantageous. The ability to secure transactions and data against common attacks in DLT systems makes IOTA suitable for tracking and verifying the authenticity of sustainable materials. This application showcases how DLT can extend beyond financial transactions to areas like supply chain management and certification processes.

### 4.4. Device Identity

Support for DIDs is mandatory in the proposed architecture. DIDs promote interoperability by providing a standardized method for creating and managing identities across different systems and platforms. This facilitates seamless interaction between various decentralized applications and services that recognize and utilize DIDs.

IOTA Identity is a Rust implementation of the DID framework, also known as Self-Sovereign Identity (SSI). The IOTA Identity library is used to generate a new DID, which results in a basic DID Document being created that includes a public key (a public–private key pair), which is coupled with the specific channel used to write the data in the IOTA network and control access to the DID document.

**Figure 3:**
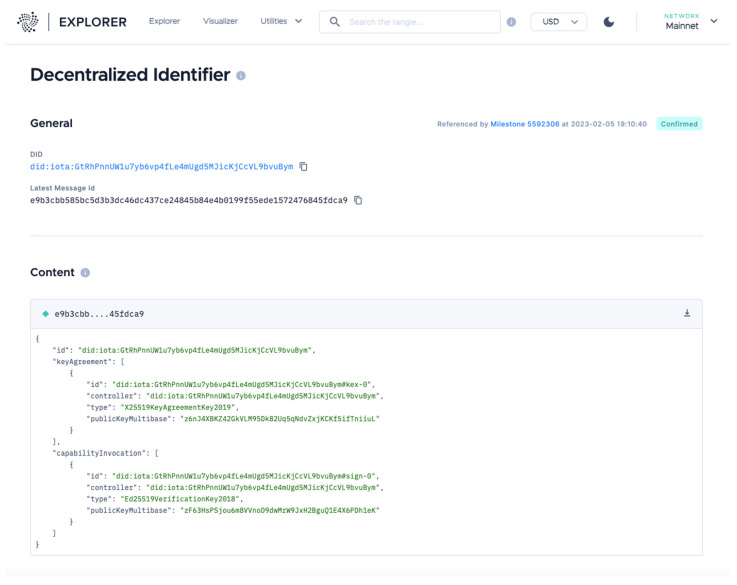
DID document publicly available on the IOTA Tangle Explorer.

Using this approach, the DID Document (Figure 3) is formatted as an integration DID message, signed using the same keypair used to generate the tag, and published to an IOTA Tangle on the index generated out of the public key used in the DID creation process. All private keys or seeds used for the *did–iota* method should be equally well protected by the users and sensors. The signing key is especially important as it controls how keys are added or removed, providing full control over the identity. Consequently, the utilization of DIDs in identifying sensors becomes imperative, ensuring a protective framework that strengthens security and fosters reliability in the system.

### 4.5. Platform Architecture

The platform serves as a nexus for aggregating data sourced from a diverse array of devices and sensors as explained above. Consequently, near real-time data transmission was implemented with publish–subscribe messaging protocol technologies such as MQTT and Kafka. All the software (versión number 1.0) and hardware infrastructure were designed in such a way that they will expedite and ease the work of end users. The whole architectural design is scalable, flexible, and ensures interoperability and easy information flow between the various components and different data concepts (e.g., real time measurements, input or output of models, economical information, etc.).

**Figure 4:**
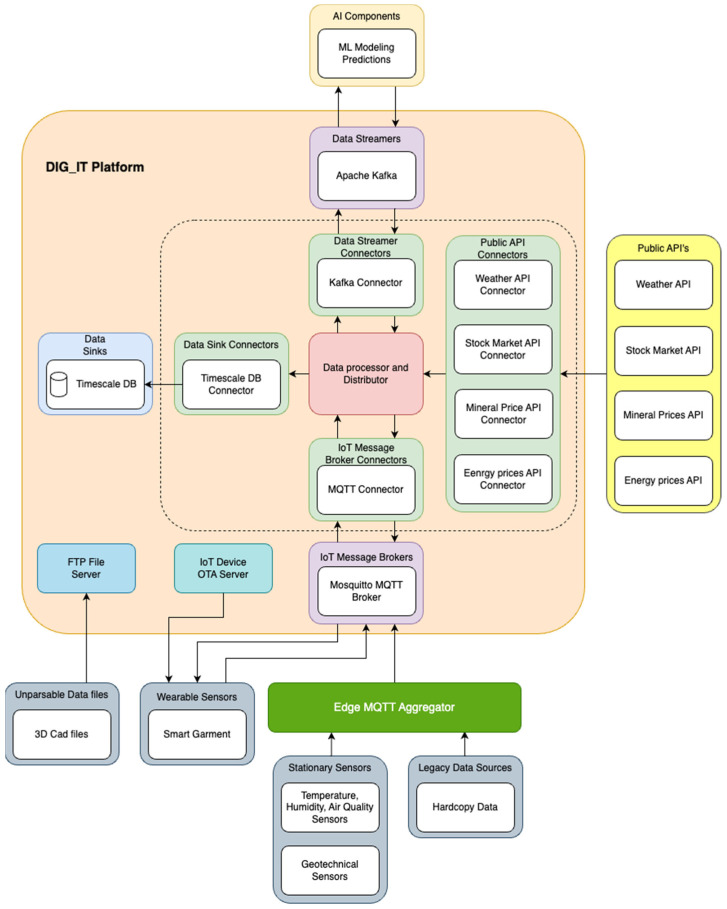
Architecture and building blocks of the data collection mining monitoring system.

The platform stores data from multiple data sources and sends them to multiple data destinations. All data are stored in a central location and can be accessed by the rest of components and users. Figure 4 summarises the high-level architecture of the platform, and its basic building blocks. The architecture represents a fundamental example of the IoT project that collects various types of data from the processes in the mines, which is why it is used as an example in this study. As shown, the platform consists of advanced components as an MQTT broker, an Apache Kafka (integrated with machine learning modelling, -ML-, and user Interface -UI-), Timescale DB, data connectors (data streamers, data sink, IoT message broker connectors), OTA servers for garment updates, and FTP servers.

The central software components of the platform are the data processor and distributor block, which data processing, harmonisation, and exportation to various destinations. Various data connectors are implemented so that data are successfully read and written by the data processor and aggregator, the various data sources, and data destinations. The main data sources are sensors data, ML models data, and data from external sources. Regarding the sensor data, there are several types of sensors and various project partners engaged in producing and sharing data. Stationary data contain data from sensors measuring temperature, humidity, PM25, PM10, concentration of CO, NO_2_, NH_3_, water level, suspended metals, etc. On the other hand, personal data gathered by using the smart garment and contain measurement obtained by wristbands, such as scalar and angular acceleration in three axes, the percentage of oxygen saturation in blood, the skin temperature, the heart rate in ppm, the electrodermal activity, the raw PPG, and the keywords from the earplug. Simultaneously, it gathers environmental (concentration of CO, NO_2_, and NH_3_), noise (noise level in dB), and UWB data, such as quality factor and location x, y, z coordinates. Other sensors’ data include geotechnical data such as data coming from piezometers and inclinometers that are forwarded to the aggregator.

**Figure 5:**
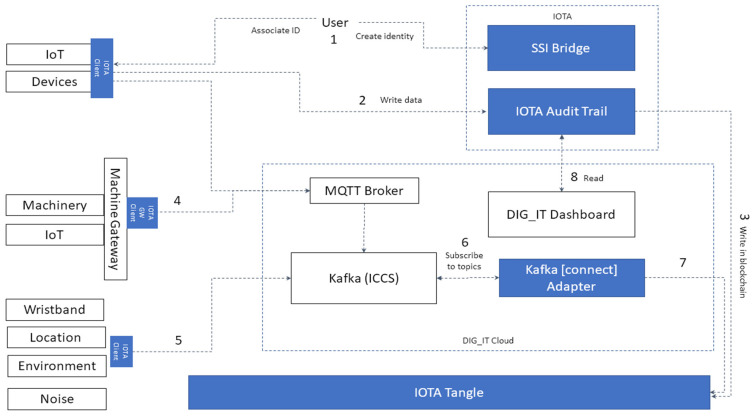
Methods for writing the data in DLT originated from various sources with different hardware and software capabilities. DIG_IT dashboard is explained below.

Keeping in mind the complexity and number of different hardware used and available data sources, as well as the inability to change the OEM firmware for some of the devices to deploy DID directly on the device, there are a number of potential solutions to writing data in the DLT:

1. Writing data directly from the device to the DLT. This approach requires libraries to be deployed on the device and this approach is conformant with end-to-end traceability. It also requires the creation of a DID for the device (Figure 5, step 1) that will write the data in the encrypted channels and in the DLT (steps 2 and 3).
2. Writing data from Gateway or Edge to the DLT. It requires Gateway running the script (Figure 5, step 4), which proxies the communication on behalf of the device.
3. Writing other sources of data collected by the Kafka event broker. The devices send the data to Kafka (Figure 5, step 5). The adapter that is subscribed to Kafka can be developed to listen to a specific channel and collect the data that will be written in the DLT.

## 5. System Dashboards

This section delineates the implementation of the envisaged architecture, culminating in the establishment of system dashboard tailored for governmental authorities, mining managers, and the general citizenry. The objective is to reproduce the scenario in which the verifier is assessing the traceability of a specific dataset.

**Figure 6:**
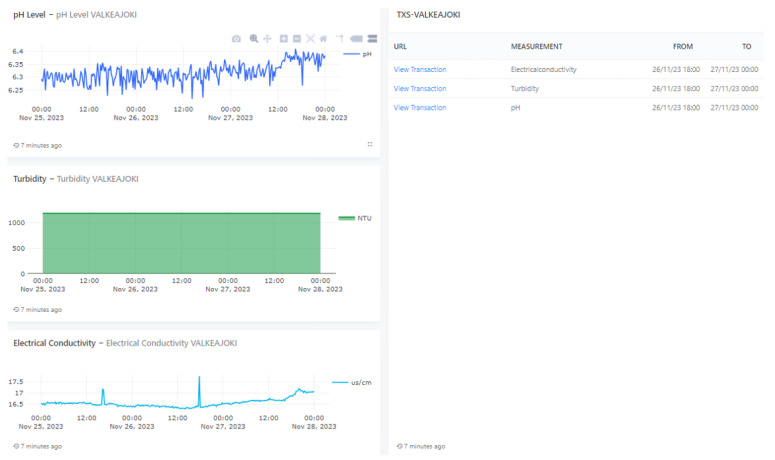
Compliance dashboard with pH, turbidity, and electrical conductivity linked to Tangle Explorer.

**Figure 7:**
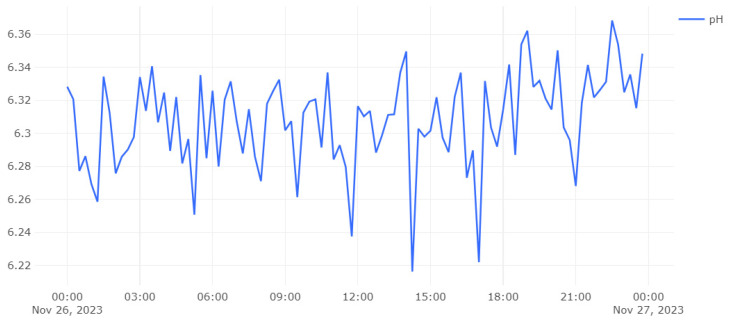
Closer look at a pH level graph.

**Figure 8:**
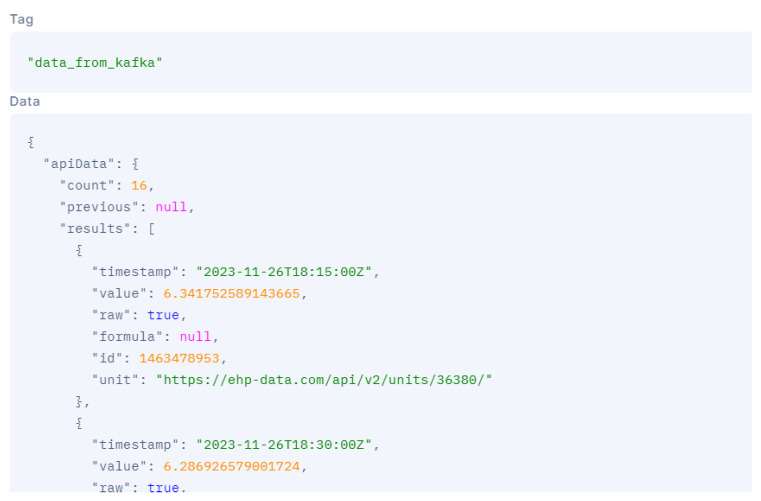
pH Level data available on the public ledger—IOTA Tangle Explorer. https://explorer.iota.org/mainnet/block/0xc2f39273ad98b033e0034cae6caf9ae39fe19506f9c27122d48c03138df3fb2c (accessed on 26 November 2023).

Figure 6 and Figure 7 show a customized dashboard in the location of Valkeajoki, where all values are displayed. Dashboards similar to this one constructed for the mentioned mine location have been developed for the other mining sites as well. A verifier can, by clicking the URL that leads to a specific dataset written in IOTA, access the given data and check using the IOTA Explorer (Figure 8) that the dataset values have not been changed. The indexation of the payload with a human-readable dataset presented over the IOTA Explorer are unencrypted so as to be available to the relevant actors, and the message tree provides visual representation of the data stored in the IOTA tree with a specific node. The message shows that the message has been indexed by the node and that data are included in the ledger.

Other dashboards (not included here) write only the data hash instead of the data itself. In this case, the hash can be presented over the dashboard using the very same approach, and the verifier would need to use the IOTA Explorer and compare the two hashes linked to a specific value. If the values or hashes do not match, the dataset has been tempered with and values have changed. Making immutable data accessible through a public dashboard enhances transparency and accountability. Government verification agencies can easily access and verify data authenticity using a unique message ID linked to the IOTA Explorer. This system ensures data integrity and prevents tampering.

The interface includes graphs and data on three different parameters: pH level, turbidity, and electrical conductivity, along with some transaction information. Here is a breakdown of each section:

- pH Level: This graph displays the pH levels over a period of several days. The pH scale, which measures how acidic or basic water is, ranges from 0 to 14, with 7 being neutral.
- Turbidity: The second graph shows the turbidity levels, which measure the clarity of the water by assessing how much particles suspended in the water scatter light.
- Electrical conductivity: The third graph shows the electrical conductivity, which indicates the water’s ability to conduct electricity.

On the right side of the image, there is a table that contains links to data that may be recorded on IOTA Explorer. Each “View Transaction” link is associated with a specific measurement (electrical conductivity, turbidity, pH) and provides a timeframe that corresponds to the date and time of the recorded data.

Figure 7 highlights one point with a pH value of 6.34175 at the specific time of 26 November 2023 at 18:15, indicating a pH value of 6.34175. As has been stated before, these timestamped data are stored in the IOTA network and can be visualized (Figure 8) when clicking in “View Transaction” for pH measure.

## 6. Discussion and Future Work

By recording key environmental metrics such as pH level, turbidity, electrical conductivity, and emissions on an immutable ledger, mining companies can provide verifiable proof of their compliance with regulatory standards. This method effectively addresses the challenges of transparency and accountability in the industry. The immutable nature of the DLT ensures that the recorded data cannot be altered, offering a permanent and trustworthy record of the mining company’s environmental impact. This is crucial in demonstrating adherence to environmental regulations and in mitigating the negative consequences of mining operations.

Furthermore, the use of blockchain allows for real-time data sharing with politicians, industrial stakeholders, and local citizens. This transparency ensures that all parties are informed of the mining activities and their environmental impact, fostering trust and collaboration between the mining industry and the affected communities.

This paper did not cover use of smart contract for the automatization of emission compliance. This is proposed in a number of studies and represents the logical next step and future work, which can be easily integrated once an approach as the one proposed in this paper is established. Integration with existing mining systems and processes also requires careful consideration to ensure a seamless transition and effective data interoperability. Moreover, regulatory frameworks and standards for blockchain implementation in the mining sector are still evolving, necessitating a harmonised approach to legal and governance aspects.

The proposed approach is not only viable in the mining industry. It can be applied to any mining materials supply chain project. Further to this, the proposed methodology goes well with the EU conflict minerals regulation (2021) [28] that came into effect as a union-wide attempt to regulate supply chains and increase transparency between conflict minerals actors.

## 7. Conclusions

It becomes evident that only advanced technologies hold the potential to comprehensively ensure compliance of the Industry 4.0 operation with the sustainable development goals. DLT, with its inherent tamper-resistant properties, serves as an ideal platform for storing and certifying these critical records. This ensures that any attempt to modify or manipulate the data is immediately detectable, maintaining the data’s credibility and integrity. These innovative safeguards not only provide a shield against unauthorised access and tampering but also establish an immutable record of data transactions.

This paper has presented secure, tamper-proof, and user-controlled identity verification and access mechanisms, an approach that lays a sound foundation for a more secure and transparent mining ecosystem. In the architecture, DIDs and the IOTA network have been used to implement encryption data records with data and hashed data anchored on the explorer. The objective was to work within labelling sustainable material certification. As an example of dashboards, the environmental parameters recorded, such as pH level, turbidity, electrical conductivity, and emissions, provide verifiable proof of their compliance with regulatory standards. Customized dashboards have been built facilitating the access to traceable information for a great variety of actors.

## Figures and Tables

**Table 1:** Comparison of DLT features.

DLT	TPS	Price (EUR)	DID	OEM Support	Consensus Mechanism
Ethereum	30	0.37 ^1^	Yes *	Yes *	PoS
Polygon	7000	0.028	Yes	No	PoS
Polkadot	1000	0.07	Yes *	No	PoS
Cardano	250–1000	0.8	Yes	No	PoS
Algorand	1000 ^2^	0.001	Yes	Yes ^3,^*	PoS
IOTA 2.0	1000 ^4^	0	Yes	Yes	DAG
EOSIO	4000	0	Yes *	No	DpoS
0Bsnetwork	NA	0.05 ^5^	No	No	NG-DpoS

* Unofficial libraries developed by third parties. ^1^ Etherscan, transaction gas fee estimator https://etherscan.io/gastracker (accessed on 14 December 2023); ^2^ Muhammed F. Esgin, Veronika Kuchta, Amin Sakzad, Practical Post-quantum Few-Time Verifiable Random Function with Applications to Algorand, Financial Cryptography and Data Security, 2021, Volume 12675, Springer; ^3^ Algorand third-party STM32 https://github.com/salvatorecorvaglia/Algorand-STM32-MPU (accessed on 07/12/2023); ^4^ N. Sealey, A. Aijaz and B. Holden, “IOTA Tangle 2.0: Toward a Scalable, Decentralized, Smart, and Autonomous IoT Ecosystem”, 2022 International Conference on Smart Applications, Communications and Networking (SmartNets), Palapye, Botswana, 2022, pp. 01–08, doi: 10.1109/SmartNets55823.2022.9994016; ^5^ 0Bsnetwork fee 0.03 EUR/KB available online: https://www.0bsnetwork.com/ (accessed on 10 November 2023).

## Data Availability

Data are contained within the article.
