# Correction: Gligoric et al. IOTA-Based Distributed Ledger in the Mining Industry: Efficiency, Sustainability and Transparency. *Sensors* 2024, *24*, 923

**DOI:** 10.3390/s24113308

**Published:** 2024-05-22

**Authors:** Nenad Gligoric, David Escuín, Lorena Polo, Angelos Amditis, Tasos Georgakopoulos, Alberto Fraile

**Affiliations:** 1 Zentrix Lab, Blockchain Development Department, Milosa Trebinjca 10, 26000 Pancevo, Serbia; 2 ITAINNOVA—Instituto Tecnológico de Aragón, C. María de Luna, 7, 50018 Zaragoza, Spain; descuin@itainnova.es (D.E.); lpolo@itainnova.es (L.P.); 3 Institute of Communications and Computer Systems: ICCS, 28is Oktovriou 42, 106 82 Athina, Greece; a.amditis@iccs.gr (A.A.); a.georgakopoulos@iccs.gr (T.G.); 4 Escuela Superior de Ingeniería y Tecnología (ESIT), Universidad Internacional de La Rioja (UNIR), 26006 Logroño, Spain; alberto.fraile@unir.net

In the original publication [1], the “Acknowledgments” section was not included. This section should include the following paragraph:

The authors wish to express their sincere gratitude for the support and collaboration of SINTEF Helgeland and, in particular, researcher Zack Klockar. This esteemed organization played a pivotal role in the success of this work, notably through its invaluable contributions to our interactions with Hannukainen and the exploration of effective communication strategies with communities. The insights and expertise offered by SINTEF Helgeland have been instrumental in advancing the objectives of this project.

The authors state that the scientific conclusions are unaffected. This correction was approved by the Academic Editor. The original publication has also been updated.
